# Finished Genome Sequence of the Indole-3-Acetic Acid-Catabolizing Bacterium Pseudomonas putida 1290

**DOI:** 10.1128/MRA.00519-19

**Published:** 2019-07-11

**Authors:** Tyler S. Laird, Johan H. J. Leveau

**Affiliations:** a Department of Plant Pathology, University of California, Davis, Davis, California, USA; University of Arizona

## Abstract

Use of indole-3-acetic acid (IAA) as a carbon, nitrogen, and energy source by Pseudomonas putida 1290 is linked to the possession of a gene cluster that codes for conversion to catechol. Here, we present the genomic context of this *iac* gene cluster, which includes genes for IAA chemotaxis/transport and catechol catabolism.

## ANNOUNCEMENT

Originally isolated from the pear phyllosphere (1), Pseudomonas putida 1290 was the first bacterium for which the ability to grow at the expense of the plant hormone indole-3-acetic acid (IAA) was linked to a gene cluster designated *iacABCDEFG-R-HI*, which allows the enzymatic conversion of IAA to catechol for further channeling into central metabolism via the beta-ketoadipate pathway (2). P. putida 1290 also exhibits positive chemotaxis toward IAA (3). To better understand the genomic context within which the *iac* gene cluster operates in this strain, we sequenced the genome of P. putida 1290.

Genomic DNA was extracted from a single-colony culture of P. putida 1290 grown overnight in LB, using a DNeasy blood and tissue kit (Qiagen, Valencia, CA) to construct a 10-kb library for PacBio RS II sequencing on 3 single-molecule real-time (SMRT) cells using P4-C2 chemistry at the UC Davis Genome Center. The total number of reads was 160,877, covering a total of 597,416,768 bp (average subread length, 3,713 bp; *N*_50_ subread length, 5,224 bp). *De novo* assembly was accomplished with Hierarchical Genome Assembly Process (HGAP) version 3 within SMRT Analysis software v2.2.0 (Pacific Biosciences) (4). Default parameters were used for this and all other software. Assembly correction was performed with Quiver and Gepard v1.30 (5), resulting in two circularized contigs, a 6,495,886-bp chromosome (G+C content, 63.14%; 62.26× coverage) and a 114,265-bp, major facilitator superfamily (MFS)-type I plasmid (6) designated pPput1290 (G+C content, 54.59%; 25.17× coverage). Pairwise average nucleotide identity values based on fastANI (7) showed the closest genomic match (87.4%) with soil isolate P. putida DRA525 (8). The plasmid most similar to pPp1290 in the PLSDB database (9) was pGRT1 from P. putida DOT-T1E (10). Gene prediction by RAST (11) revealed 6,048 coding sequences, 77 tRNA genes, and 20 rRNA genes on the chromosome and 123 coding sequences on pPp1290.

We identified an open reading frame (ORF), directly upstream of the *iacABCDEFG-R-HI* gene cluster in P. putida 1290, coding for a LysR-type transcriptional regulator, a partial duplication of the *iac* gene cluster (*iacCDF*), genes predicted to code for an OprD-like outer membrane protein, an ATP-binding protein belonging to the ABC family of transporters, and an MFS-type transporter similar to IacT1 from Paraburkholderia phytofirmans PsJN, implicated in the transport of an IAA degradation pathway intermediate (12). Also upstream of the *iac* gene cluster, an ORF predicted to code for a methyl-accepting chemotaxis protein possibly involved in the chemotaxis of P. putida toward IAA (3) and a *catR-catBCA* gene cluster for catechol catabolism (3) were found. Interestingly, the *iac* gene cluster appears to be part of an approximately 30-kb genomic island predicted by the IslandPick algorithm in IslandViewer v4 (13) and encompassing multiple genes for the utilization of plant-derived compounds other than IAA, including methylamine (14), phenylacetaldehyde (15), and opines (16). The presence of *aldA* and *aldB* homologs (17) on the chromosome is consistent with IAA production reported for P. putida 1290 (2). Plasmid pPp1290 harbors multiple insertion sequence (IS)-like elements, as well as genes related to aromatic compound degradation, stress tolerance, fluoride efflux, and conjugal transfer.

### Data availability.

The P. putida 1290 chromosome and plasmid sequences are available under GenBank accession numbers CP039371 and CP039372, respectively. Raw reads from the three SMRT cells are available under the SRA accession number SRP195595.
